# Elucidation of Sigma Factor-Associated Networks in *Pseudomonas aeruginosa* Reveals a Modular Architecture with Limited and Function-Specific Crosstalk

**DOI:** 10.1371/journal.ppat.1004744

**Published:** 2015-03-17

**Authors:** Sebastian Schulz, Denitsa Eckweiler, Agata Bielecka, Tanja Nicolai, Raimo Franke, Andreas Dötsch, Klaus Hornischer, Sebastian Bruchmann, Juliane Düvel, Susanne Häussler

**Affiliations:** 1 Institute for Molecular Bacteriology, TWINCORE GmbH, Center for Clinical and Experimental Infection Research, a joint venture of the Hannover Medical School and the Helmholtz Centre for Infection Research, Hannover, Germany; 2 Department of Molecular Bacteriology, Helmholtz Centre for Infection Research, Braunschweig, Germany; 3 Department of Chemical Biology, Helmholtz Centre for Infection Research, Braunschweig, Germany; University of Maryland, UNITED STATES

## Abstract

Sigma factors are essential global regulators of transcription initiation in bacteria which confer promoter recognition specificity to the RNA polymerase core enzyme. They provide effective mechanisms for simultaneously regulating expression of large numbers of genes in response to challenging conditions, and their presence has been linked to bacterial virulence and pathogenicity. In this study, we constructed nine his-tagged sigma factor expressing and/or deletion mutant strains in the opportunistic pathogen *Pseudomonas aeruginosa*. To uncover the direct and indirect sigma factor regulons, we performed mRNA profiling, as well as chromatin immunoprecipitation coupled to high-throughput sequencing. We furthermore elucidated the *de novo* binding motif of each sigma factor, and validated the RNA- and ChIP-seq results by global motif searches in the proximity of transcriptional start sites (TSS). Our integrated approach revealed a highly modular network architecture which is composed of insulated functional sigma factor modules. Analysis of the interconnectivity of the various sigma factor networks uncovered a limited, but highly function-specific, crosstalk which orchestrates complex cellular processes. Our data indicate that the modular structure of sigma factor networks enables *P*. *aeruginosa* to function adequately in its environment and at the same time is exploited to build up higher-level functions by specific interconnections that are dominated by a participation of RpoN.

## Author Summary

*Pseudomonas aeruginosa* is well known for its high adaptability to a large range of environmental conditions, including those encountered within the human host. Transcription initiation represents a major regulatory target which drives versatility, and enables bacterial adaptation to challenging conditions and expression of virulence and pathogenicity. In bacteria, this process is largely orchestrated by sigma factors. Here, we performed an integrative approach, and by the combined use of three global profiling technologies uncovered the networks of 10 alternative sigma factors in the opportunistic pathogen *P*. *aeruginosa*. We demonstrate that these networks largely represent self-contained functional modules which exhibit a limited but highly specific crosstalk to build up higher-level functions. Our results do not only give extensive information on sigma factor binding sites throughout the *P*. *aeruginosa* genome, but also advance the understanding of sigma factor network architecture which provides bacteria with a framework to function adequately in their environment.

## Introduction

The ability to maintain homeostasis even in changing environments and under extreme conditions is one of the key traits of living organisms. *Pseudomonas aeruginosa* is a ubiquitous gram-negative bacterium that can be distinguished by its exceptional high capability to adapt and survive in various and challenging habitats [1]. The reason for the remarkable ecological success of *P*. *aeruginosa* can be attributed to its large metabolic versatility and environment-driven flexible changes in the transcriptional profile. *P*. *aeruginosa* is not only an adaptive environmental bacterium but also an important opportunistic pathogen which exhibits an extremely broad host range [2,3]. It is the causative agent of acute and chronic, often biofilm-associated, infections particularly in the immunocompromized host and cystic fibrosis patients [4–6].

Genome sequencing of *P*. *aeruginosa* reference strains revealed a large genome with highly abundant global regulators and signaling systems that form a complex and dynamic regulatory network responsible for phenotypic adaptation and virulence [7–9]. Among transcriptional regulators, sigma factors are of exceptional importance as they confer promoter recognition specificity to the RNA polymerase [10,11]. They are essential for transcription initiation [12] which is the key step in gene regulation [13]. Alternative sigma factors and in particular extracytoplasmic function (ECF) sigma factors can provide effective mechanisms for simultaneously regulating expression of large numbers of genes in response to challenging conditions [14]. *P*. *aeruginosa* encodes more than 25 sigma factors most of which, including one strain-specific sigma factor, were reviewed in 2008 [14]. Among them are at least 21 ECF sigma factors [15] [16] whose presence has been linked to bacterial virulence and pathogenicity [15,17–19].

The advent of microarray technology has promoted the elucidation of bacterial genetic regulatory networks involved in adaptation to various environmental stresses and physiological processes [20]. Subsequently, the combination of DNA microarray technology and chromatin immunoprecipitation (ChIP-chip) offered the opportunity to distinguish direct binding sites of transcription- and sigma-factors from those bound indirectly [21–23]. With these valuable tools at hand, sigma factors gained greater attention and their impact on gene expression has become a major research focus [19,24–29]. In this study, we constructed strains expressing his-tagged sigma factors *in trans* and/or sigma factor deletion mutant strains and performed mRNA profiling as well as chromatin immunoprecipitation coupled to high-throughput sequencing to uncover the direct and indirect regulons of 10 alternative sigma factors in *P*. *aeruginosa*. Our results contribute to a deeper understanding of global gene regulation in bacteria and provide a reliable scaffold for the elucidation of the transcriptional regulatory network of the important pathogen *P*. *aeruginosa*.

## Materials and Methods

### Strains and plasmids

Sigma factor genes were amplified by PCR using a forward primer harboring a ribosomal binding site and the ATG start codon and a reverse primer with the stop codon TGA (S1 Table). PCR products were introduced into pJN105 [30] under control of P_BAD_ resulting in pJN105-RBS-σ. For ChIP-seq experiments pJN105-RBS-σ-8xhis was constructed using a reverse primer additionally encoding for an 8xHis-tag and for bioluminescence assays selected sigma factor target promoters were ligated into pBBR1-MCS5-TT-RBS-*lux* [31]. Vectors were transferred into respective *P*. *aeruginosa* PA14 strains by electroporation as previously described [32]. The PA14Δσ::Gm^r^ deletion mutants were constructed according to a modified protocol using overlap extension PCR [33]. The gene replacement vector pEX18Ap [34] was modified by inverse PCR to remove the coding sequence for 5S rRNA. In addition, the resulting vector pEX18Ap2 encompasses a novel MCS established by primer extension. Regions up- and downstream of the sigma factor gene were amplified by PCR (S1 Table). The primer Mut-σ-up-RV and Mut-σ-down-FW harbored complementary sequences coding for three shifted stop codons and a KpnI restriction site (XmaI for *rpoN*). The two corresponding PCR products were fused in a second PCR and the obtained fragment was introduced in pEX18Ap2 resulting in pEX18Ap2-up-σ-down-σ. pEX18Ap2-up-σ-Gm-down-σ vectors were produced by ligation of a FLP-excisable gentamicin cassette amplified from pUC18-mini-Tn7T-Gm-*lacZ* into pEX18Ap2-up-σ-down-σ. Single crossovers in PA14 were selected on gentamicin. Counter-selection in LB low salt supplemented with sucrose resulted in PA14Δσ::Gm^r^. Counter-selection for PA14Δ*sigX*::Gm^r^ was performed in BM2 [35] and PA14Δ*rpoN*::Gm^r^ in LB supplemented with 1 mM glutamine. The gentamicin cassette was excised from PA14Δ*sigX*::Gm^r^ and from PA14Δ*rpoN*::Gm^r^ using the FLP expression vector pFLP3 [36] to obtain PA14Δ*sig* and PA14Δ*rpoN*.

### mRNA profiling

RNA was prepared from PA14 wild-type, PA14Δσ:Gm^r^, PA14 (pJN105) and PA14 (pJN105-RBS-σ) in two independent experiments each containing a pool of three individual main cultures (in 10 ml medium at 37°C). 0.5% L-arabinose was added to PA14:pJN105-RBS-σ and the corresponding control PA14:pJN105 for at least 35 min. To maximize expression of the sigma factor dependent regulons the strains were cultivated under conditions previously shown to induce the activity of the various sigma factors. Therefore, PA14 (pJN105-RBS-*fliA*) was harvested in the exponential phase (OD_600_ = 1.1), PA14 (pJN105-RBS-*rpoS*) and PA14 (pJN105-RBS-*rpoN*) were cultivated to the early stationary phase (OD_600_ = 2.0). PA14 (pJN105-RBS-*algU*) was grown to an OD_600_ of 2.3 and exposed to 50°C for 5 min. PA14 (pJN105-RBS-*rpoH*) was grown at 28°C up to an OD_600_ of 1.4–1.5 including 35 min induction of *rpoH* expression and was exposed to 42°C for 5 min. PA14 (pJN105-RBS-*sigX*) was cultivated in low osmolarity LB containing 8 mM NaCl. PA14 (pJN105-RBS-*pvdS*), PA14 (pJN105-RBS-*fpvI*), PA14 (pJN105-RBS-*fecI*) and PA14 (pJN105-RBS-*fecI2*) were exposed to iron starvation (growth in 50% LB to OD_600_ of 1.5, incubation with the iron-chelating agent 2,2’-bipyridyl (200 μM) for 70 min). PA14Δσ::Gm^r^ deletion mutants were cultivated as the sigma factor *in trans* expressing strains. PA14Δ*rpoN*::Gm^r^ was also cultivated under nitrogen-limitation in BM2 containing 0.1% casein amino acids as sole nitrogen source to an OD_600_ of 1.2 and the growth-impaired PA14Δ*sigX* strain was grown under low osmolarity condition to the same OD as the corresponding PA14 wild-type strain. RNA extraction, cDNA library preparation and Illumina sequencing were performed as previously described [37]. In brief, cells were harvested after addition of RNA protect buffer (Qiagen) and RNA was isolated from cell pellets using the RNeasy plus kit (Qiagen). mRNA was enriched (MICROBExpress kit (Ambion)) fragmented and ligated to specific RNA-adapters containing a hexameric barcode sequence for multiplexing. The RNA-libraries were reverse transcribed and amplified resulting in cDNA libraries ready for sequencing. All samples were sequenced on an Illumina Genome Analyzer II-x in the Single End mode with 36 cycles or on a HiSeq 2500 device involving 50 cycles.

### Quantification of gene expression

Sequences were mapped to the PA14 genome using stampy [38] with default settings and the R package DESeq [39] for differential gene expression analysis. Differentially expressed genes were identified using the *nbinomTest* function based on the negative binomial model after pre-filtering by overall variance [40]. The Benjamini and Hochberg correction was used to control the false-discovery rate at 0.05 to determine the list of regulated genes [39]. The quality control output in PDF format is available for download as part of the supplementary information accompanying GEO dataset. Genes were identified as differentially expressed if they fulfilled the following criteria: *i)* an at least three-fold down-regulation in the sigma factor mutant as compared to the corresponding wild-type strain or an at least three-fold up-regulation in the strains expressing the sigma factor *in trans* as compared to the cognate empty vector control strain and *ii)* the Benjamini-Hochberg corrected P value was smaller than 0.05 with the exception of PA14Δ*fpvI*:Gm^r^ and PA14Δ*fecI*:Gm^r^ whose cut-off values were set to a fold change of at least 2 using the uncorrected P value. To appraise sigma factor competition, we determined also the negative impact of the expression of the sigma factor *in trans* on genes and considered genes which were at least three-fold down-regulated with a maximal P value of 0.05.

### Hierarchical clustering

We computed the pair-wise Pearson correlation between the log_2_-normalized read counts (*nRPKs*) of all but the rRNA/tRNA genes in PA14 using data from all transcriptome replicates generated for a given condition and sigma factor. We performed hierarchical clustering of the genes in the resulting expression matrix (10 alternative transcription factors * 2 replicates * knockout/in-trans expressing conditions) by progressively grouping them: at each step of the iterative algorithm the two genes or gene clusters that have the smallest distance were merged to form a new cluster, and two branches of a growing tree were joined. The lengths of the branches are equal to the half of the distance between two genes or gene clusters. We used the average linkage rule; this means that the distance between two clusters is computed as the mean of all the distances between the genes in the first cluster and the genes in the second cluster. All calculations were performed in R using the *hclust* function.

### Chromatin immunoprecipitation followed by deep sequencing

ChIP-seq was applied to four 20 ml cultures (with pooling of two individual cultures) of PA14(pJN105-RBS-σ-8xHis) and PA14(pJN105) as a control. ChIP-seq samples were treated under the same condition as described for mRNA profiling with the exception of PA14(pJN105-RBS-rpoH-8xHis) which was exposed to a heat-shift from 37°C to 42°C. Following treatment with 0.5% formaldehyde and glycine cells were harvested, washed and suspended in 0.5 ml of lysis buffer. DNA was fragmented to an average size of 200 to 250 bp and subjected to chromatin immune-precipitation with 15 μl of anti-6xHis tag antibody (ab9108; abcam) overnight at 4°C. Following an incubation step with 1 μl of RNase A (100 mg ml^−1^) and proteinase K (20 mg ml^−1^) immunoprecipitated DNA was recovered using a QIAquick PCR Purification kit (Qiagen) and subjected to a modified linear DNA amplification (LinDA) protocol [41]. For next generation Illumina sequencing, up to 50 ng of DNA was used in a TruSeq DNA sample preparation kit (Illumina) according to the low-throughput protocol. ChIP-seq data was analyzed by removing adapter sequences using the fastq-mcf script that is part of the EA-utils package [42]. Reads were trimmed allowing for minimal quality of 10 at their ends. We used the Bowtie aligner [43] to map the reads. Model-based analysis of ChIP-seq [44] was applied for peak detection using a P value cut-off value of 0.05 and shift size 30 for the peak modeling, making use of the relevant control samples. Details on the promoter hits from the individual replicates are available in S5 Table. Promoter hits were considered significant when they were detected in both ChIP-seq replicates with an enrichment factor of at least 3 and a P value of less than 0.01. Statistical analysis of the obtained candidates was performed to assess the number of false positives and the corresponding P value according to the hypergeometric test in R using the *phyper* command.

### Chromatin immunoprecipitation followed by microarray analysis

ChIP results using an anti-RpoS polyclonal antibody followed by microarray analysis was included in this study [45]. DNA was purified and amplified and approximately 7.5 μg of amplified DNA from the control and the RpoS ChIPs were sheared to a fragment size of 50 to 500 bp and terminally labeled using the GeneChip WT double stranded DNA terminal labeling kit (Affymetrix). The biotin-labeled DNA was hybridized to an Affymetrix *P*. *aeruginosa* genome chip as described previously [46]. Enrichment of hybridization signals was calculated with the Tiling Analysis Software (TAS, Affymetrix) from two independent RpoS ChIPs compared to two independent mock ChIPs (bandwidth parameter was set to 150 bp). For each gene, the promoter region was defined as the sequence from -300 to -1 bp based on the PAO1 annotation [47]. Threshold levels for significantly enriched promoter region were log_2_ enrichment factor of at least 0.5 with P value less than 0.05 across at least 70 bp DNA segments.

### Transcription start site sequencing

We selectively sequenced the 5’-ends of primary transcript samples of PA14 cultivated under six different growth conditions: transitional phase between exponential and stationary growth phase (OD_600_ = 1.5–2.0), exponential phase (OD_600_ = 0.5–1.5), heat shock at 42°C and 50°C, low osmolarity and iron depletion. Total RNA was extracted and treated with terminator exonuclease as described previously [48] to remove processed and incomplete transcripts (including tRNA and rRNA). The remaining primary transcripts were ligated to 5’-RNA adapters using T4 RNA ligase and subsequently reverse transcribed using SuperScript III (Invitrogen) reverse transcriptase with a modified RT-N_8_ primer containing an octameric random sequence at its 3’-end (5‘-GCT GAA CCG CTC TTC CGA TCT NNN NNN NN -3‘). The resulting cDNA contains random 5’-ends while the 3’-ends conserve the 5’-ends of the original RNA species. A PCR with primers equivalent to the Illumina Paired End Primers was performed on the cDNA yielding double-stranded cDNA libraries that were subsequently sequenced on an Illumina Genome Analyzer IIx.

### TSS categorization

The sequenced 5´end primary transcript data were clipped to remove low quality sequences and adapters and were subsequently mapped to the reference genome of *P*. *aeruginosa* PA14 using bowtie [43]. In order to detect putative TSS, read counts were normalized by the total number of reads present in each sample obtaining Reads Per Million mappable reads (RPM) and TSS were detected by selecting sites that exceeded 10 RPM in any of the 8 samples grown under six different environmental conditions. TSS separated by less than 3 base pairs were merged, and the position of the TSS having the highest RPM was set as position of the putative TSS. This resulted in a final list of 5583 TSS that were classified as described previously [37]: i) promoter TSS (3520 sites corresponding to 2309 genes), if they were detected up to 500 base pairs upstream of a gene on the same strand, ii) intragenic TSS (1709 sites), if they were detected within the margins of a gene on the same strand, iii) antisense TSS (1027 sites), if they were detected within the margins of a gene on the opposite strand or iv) orphan TSS (1156), if they were not detected within or upstream of a gene on the same strand (in other words, neither promoter nor intragenic TSS). The 2309 genes having promoter TSS had additional 1211 alternative TSS (2309+1211 = 3520 TSS). Thus, many genes (739) contained more than one alternative TSS.

### Computational prediction of transcriptional units

We defined TUs by applying a combination of three independent criteria on all annotated genes of the PA14 genome: *i)* a TSS was detected in our TSS-Seq approach within 500 bp upstream of the gene on the same strand, *ii)* the immediate upstream gene on the same strand shows at least a two-fold difference in the median gene expression across the 47 transcriptomes of *P*. *aeruginosa* PA14 wild type, and *iii)* a gene is predicted to be the first (or only) gene on an operon in the DOOR database [49]. The DOOR database includes operon predictions based on intergenic distance, neighborhood conservation, phylogenetic distance, information from short DNA motifs, similarity score between GO terms of gene pairs and length ratio between a pair of genes [50] and was found to predict operons in *E*. *coli* with 93.7% accuracy [51]. To increase the accuracy of our overall TU prediction we employed a conservative approach and assigned TUs only if both criteria *i)* and *ii)* or criterion *iii)* were fulfilled, resulting in 3687 TUs (S4 Table). Most of these TUs were already included in the DOOR database except 159 TUs that were only positive in criteria *i)* and *ii)*. For 2025 of those 3687 TUs we were able to detect the TSS positions experimentally. A large fraction, 2499 (67.7%) of those 3687 TUs were singleton operons, further 657 TUs contained 2 genes on the operon. Wurtzel et al. [52] previously defined 3794 TUs (2117 of which were detected experimentally). We furthermore analyzed the overlap of 1381 TSS which shared a respective TU as determined previously [52] and in this study. Whereas 69% of them (958 TSS) were separated by < 2bp, only 15% of them (205 TSS) were positioned 50bp or more apart from each other on the genomic coordinate.

### *De novo* motif elucidation and global motif scans

In general, sigma factor binding motif was identified by applying the MEME suite [53] on promoter regions (300 bp upstream of start codons) whose respective genes *(i)* showed at least a three-fold sigma factor-dependent down-regulation in PA14Δσ::Gm^r^ and at least a three-fold sigma factor-dependent up-regulation in PA14 (pJN105-RBS-σ) or alternatively a more than ten-fold down-regulation in PA14Δσ::Gm^r^ only or a more than ten-fold up-regulation in PA14 (pJN105-RBS-σ) only and *(ii)* were defined to be the first gene of a transcriptional unit. The SigX motif is based on genes which are the first gene of a TU, show a differential expression of at least 3 and whose promoters were enriched at least three-fold (P value less than 0.01) in both ChIP-seq replicates. For RpoD the TOP 3 motifs were elucidated selecting promoters which are enriched at least 5-fold in both ChIP-seq replicates (P value less than 0.001) and whose genes are the first gene of a TU and whose gene expression was not affected by alternative sigma factors. General parameters were selected as followed: occurrence (0 or 1 per sequence), number of sites (minimum, 7) and activated DNA option ‘search given strand only’. The motif width was adapted to each sigma factor. Furthermore, a background Markov model was supplied. The obtained motif was forwarded to FIMO [54] to identify putative sigma factor binding sites in all promoter regions across the PA14 genome. Promoter hits with a P value less than 0.0005 were regarded as significant and were listed in S5 Table.

### Definition and functional profiling of primary sigma factor regulons

A gene was defined to be a member of the primary sigma factor regulon if it fulfilled at least two of the following three criteria: *i)* it exhibited sigma factor-dependent regulation of expression, *ii)* its promoter was enriched in ChIP-seq experiments and *iii)* its promoter contained a sigma factor binding motif.

Since RpoD is an essential gene no deletion mutant could be constructed and *in trans* expression of RpoD only led to up-regulation of three genes—probably due to high abundance of RpoD in the cell, Thus, no RNA-seq data were available to describe the impact of RpoD of the global transcriptional profile. We therefore considered those genes that were not differentially regulated by any of the tested alternative sigma factors as belonging to the primary RpoD regulon, however, only if they were either found in the RpoD ChIP-seq approach or harbored an RpoD motif.

Statistical significance of these primary regulon members was checked by performing a hypergeometric test using the *phyper* command in R on the intersections ChIP-seq/RNA-seq, RNA-seq/motif search, and ChIP-seq/motif search (S7 Table and S3 Fig.). To include not only first genes but all genes of identified transcriptional units from S4 Table, downstream genes were added, if the first gene met the criteria indicated above. These final sets of genes (S6 Table) were functionally characterized using the PseudoCAP annotation [55]. To further improve this profiling, the PseudoCAP PA14 annotation was updated by adding the PseudoCAP classes of PAO1 homologs to corresponding PA14 genes. Over- or underrepresentation of each PseudoCAP category was calculated by comparing normalized PseudoCAP category experimentally detected and normalized PseudoCAP category annotated as previously described [19]. The enriched categories and their P values obtained using a hypergeometric test are listed in S8 Table.

### Bioluminescence assay

For each bioluminescence assay, three independent experiments were performed and each experiment included pooling of three biological replicates. Reporter strains (S1 Table) harboring selected sigma factor target promoter fused to the *luxCDABE* cassette of *Photorhabdus luminescens* were grown under same conditions as described under mRNA profiling. Bioluminescence of 200 μl bacterial suspension was measured in a black 96-well microtiter plate with a transparent and flat bottom. In parallel, cell density was determined using a standard photometer. Relative light units (RLU) were normalized to the optical density at a wavelength of 600 nm and the arithmetic average was calculated. Next, the average bioluminescence over the three independent assays was calculated and compared to the bioluminescence of the respective control reporter strain, e. g. the reporter construct in the corresponding sigma factor mutant strain or the PA14 wild-type strain harboring the empty vector. The standard error was determined and the statistical significance was examined using the two-tailed Student’s t-test assuming unequal variances.

### Additional information

The raw and processed data have been deposited in the Gene Expression Omnibus (GEO) database (accession numbers GSE54997 and GSE54998 united under SuperSeries GSE54999). The short read data is available through the GEO interface under projects SRP037770 and SRP037771.

## Results

### Sigma factor-associated networks in *P*. *aeruginosa*

In this study, we aimed at deciphering the regulons of alternative sigma factors and to quantify their relative contribution to the overall transcriptome plasticity in the opportunistic pathogen *P*. *aeruginosa*. We therefore amended our previously published data on the impact of the alternative sigma factor SigX [19] on the global transcriptional profile in the type strain PA14 and further expressed the his-tagged alternative sigma factors AlgU, FliA, RpoH, RpoN, RpoS, PvdS, FpvI, FecI and FecI2 *in trans* (S1 Table). We also inactivated these alternative sigma factors (with the exception of RpoH and FecI2) and recorded transcriptional profiles (S2 and S3 Table) under growth conditions that are expected to support sigma factor dependent gene expression (see Materials and Methods for details). We observed activity of all sigma factor target promoters (not done for the FpvI, FecI and FecI2 sigma factor targets which are known to respond to low iron medium conditions) under the selected experimental conditions. This activity was strictly dependent on the presence of the respective alternative sigma factor in the reporter strain (AlgU, FliA, RpoN, RpoS, SigX) or on the presence of inducing conditions (RpoH, PvdS) (S1 Fig.).

**Fig 1:**
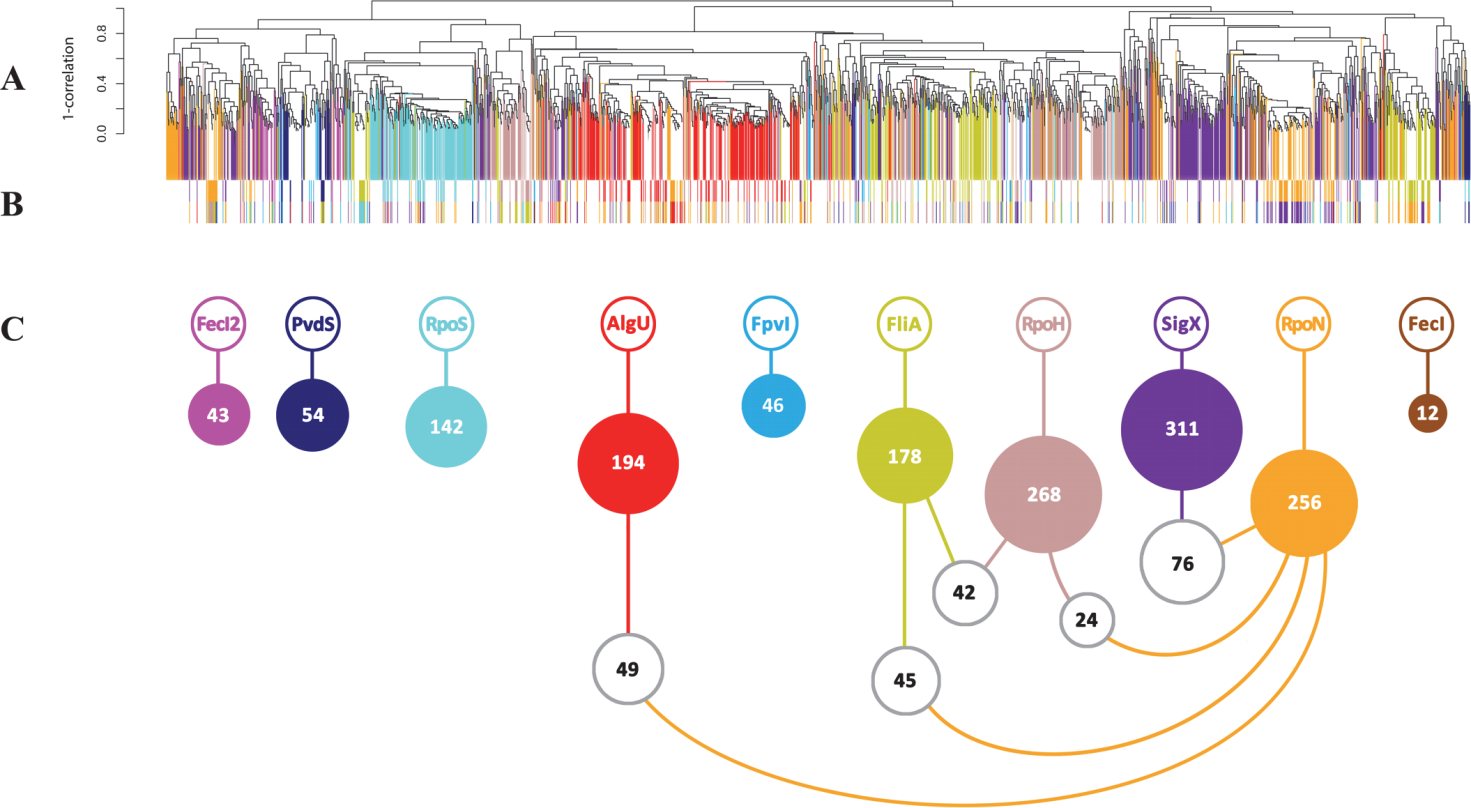
Conserved and diverged co-expression patterns of differentially regulated genes upon (hyper-) presence and/or absence of sigma factors. (A) Hierarchical clustering based on Pearson correlation coefficients. 1504 genes (67.8%, vertically colored) were differentially regulated due to *in trans* expression and/or inactivation of a single sigma factor. Genes regulated by more than one sigma factor are shown in white. (B) The expression levels of 471 genes (21.2%) were influenced by two sigma factors (indicated by the two colored bars). (C) White balls highlight the (the number of) genes that are affected by the five most frequent sigma factor combinations.

Overall, 491 genes were up-regulated in response to (hyper-) presence and down-regulated in the absence of at least one alternative sigma factor (Fig. 1A). Additional 1195 genes were up-regulated in response to (hyper-) presence and 532 were down-regulated in the absence of at least one alternative sigma factor. Interestingly, the majority of 1504 out of the overall 2218 genes (67.8%) were differentially regulated due to *in trans* expression and/or inactivation of only one single sigma factor (colored bars in Fig. 1A). The finding that many genes are exclusively affected by only one alternative sigma factor indicates that the alternative sigma factor regulons are distinct functional modules and that they have only a limited overlap at the level of transcription. Nevertheless, there was also shared regulation: the expression level of 471/2218 genes (21.2%) was influenced by two sigma factors (white bars in Fig. 1A, colored bars in Fig. 1B) and 243/2218 genes (11%) were influenced by more than two sigma factors (S2 and S3 Table). Thus, in addition to the detection of largely isolated regulons (Fig. 1A), transcriptional profiling also uncovered a co-ordinated gene expression pattern in which many genes were affected by distinct sets of alternative sigma factors (Fig. 1B and C and Table 1).

**Table 1:** Quantitative analysis of sigma factor regulons uncovered by mRNA profiling.

Regulon size^a^	610	600	520	440	372	219	184	159	129	57
Sigma factor	SigX	RpoN	RpoH	FliA	AlgU	RpoS	FpvI	PvdS	FecI2	FecI
FecI	2	0	2	0	0	1	1	1	2	**12**
FecI2	3	0	1	4	1	0	4	2	**43**	2
PvdS	11	3	1	7	4	3	6	**54**	2	1
FpvI	13	7	10	8	0	0	**46**	6	4	1
RpoS	18	12	7	14	2	**142**	0	3	0	1
AlgU	15	49	21	10	**194**	2	0	4	1	0
FliA	23	45	42	**178**	10	14	8	7	4	0
RpoH	16	24	**268**	42	21	7	10	1	1	2
RpoN	76	**256**	24	45	49	12	7	3	0	0
SigX	**311**	76	16	23	15	18	13	11	3	2

The total sizes of the individual regulons are listed at the top. Values in the colored cells show the numbers of genes regulated uniquely by the sigma factors. The intersection values show how many genes were cross-regulated by two sigma factors.

^a^ The regulon size is the sum of all genes found to be significantly (fold change ≥ 3, corrected P value ≤0.05, see Materials and Methods for exceptions) regulated upon inactivation and/or *in trans* expression of the sigma factor.

### Robustness of overall gene expression to shifts of sigma factor levels

In this study, transcriptional profiling was performed either in the absence and or *in trans* expression of the various alternative sigma factors to improve the elucidation of the primary and complete sigma factor regulons. This strategy was proven valid for numerous sigma factors in *P*. *aeruginosa* [15,56–58]. However, since there is a limited amount of RNA polymerase in the cell, we analyzed whether sigma factor expression might negatively impact the global gene expression profile under our experimental conditions. Overall we found 644 genes (10.9% of the whole genome) that were three-fold down-regulated upon *in trans* expression of any one of the ten alternative sigma factors, 169 genes that were negatively affected by two of the ten sigma factors and only 85 genes affected by more than two sigma factors. These results indicate that although the expression of a distinct set of genes might be affected by sigma factor competition for the RNA polymerase, there is no notable alternative sigma factor competition on a global scale under our experimental conditions. It seems that in *P*. *aeruginosa* competition of alternative sigma factors for a limiting amount of RNA polymerase does not play a general role and indicates robustness of overall gene expression to shifts of alternative sigma factor levels.

### The primary sigma factor regulome

To define the primary regulons of the *P*. *aeruginosa* sigma factors we complemented our transcriptome data with chromatin immunoprecipitation followed by high-throughput sequencing (ChIP-seq) and in case of RpoS with ChIP-chip experiments. This allows the differentiation of direct from indirect sigma factor-dependent regulation of genes. We constructed variants of the housekeeping sigma factor RpoD and (in addition to SigX [19]) the nine alternative sigma factors fused to an octahistidine-tag and sequenced sigma factor bound genomic DNA.

**Fig 2:**
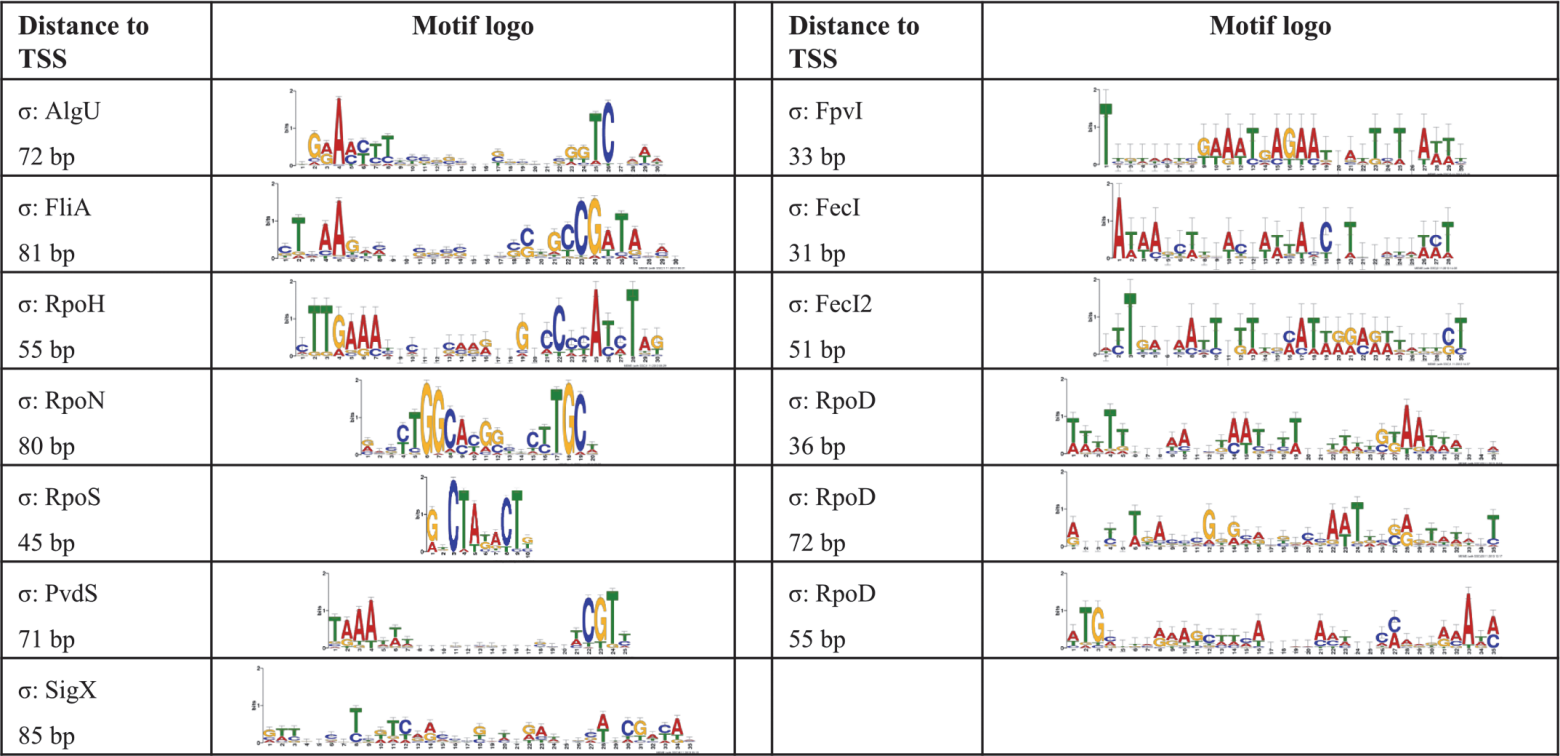
*De novo* elucidated sigma factor binding motifs. The MEME suite [53] was used to elucidate the sigma factor binding motifs (see Material and Methods section for details). For each sigma factor the motif representation is depicted and the average distance to the TSS is given.

**Fig 3:**
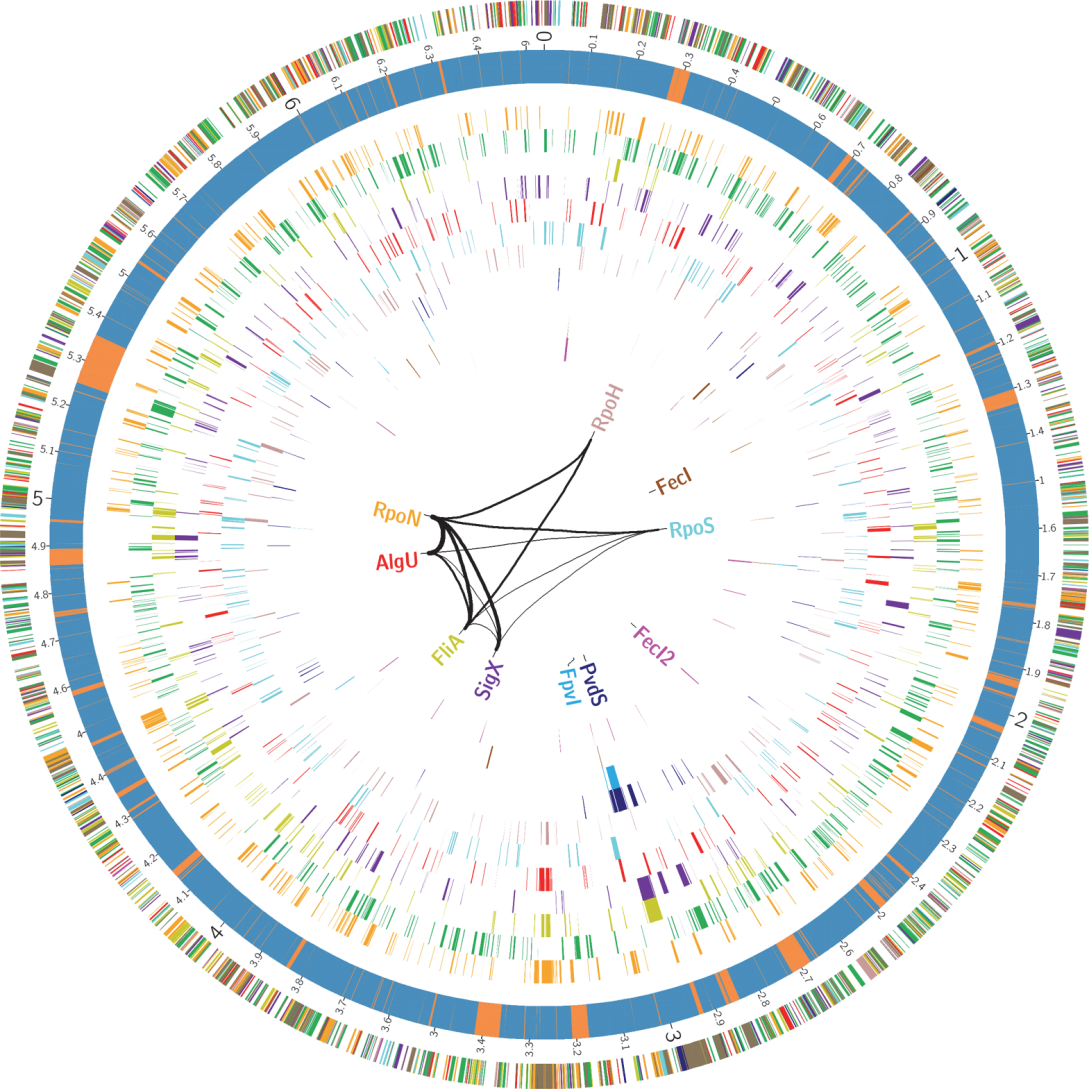
The *P*. *aeruginosa* strain PA14 sigma factor regulome. The outer circle summarizes the distribution of the 2553 genes belonging to the primary PA14 sigmulome, including RpoD (depicted in green).Those genes that belong to more than one primary sigma factor regulon are indicated in brown. The second circle represents the PA14 core (blue) and accessory (orange) genome as previously defined [90]. The inner eleven circles highlight the genes associated with individual sigma factor primary regulons. The innermost diagram depicts the TOP 12 direct cross talks between the sigma factors. The figure was generated using Circos [91]. A high-resolution interactive image is available at https://bactome.helmholtz-hzi.de/.

In order to define transcriptional start sites (TSS) and to predict the transcriptional units (S4 Table), we selectively sequenced the 5’-ends of primary transcript samples of PA14 cultivated under six different growth conditions (as outlined in material and methods). This served as the basis for elucidating the *de novo* binding motif of each sigma factor [53] (Fig. 2). We used the MEME suite (27) on those promoter regions whose respective genes exhibited an alternative sigma factor dependent regulation of expression and which were upstream of the first gene within a transcriptional unit (see Material and Methods section for details). We were able to generate a *de novo* sequence logo for each of the ten alternative sigma factors. Furthermore, in 76.4% of the genes that harbored a sigma factor binding motif and for which a TSS could be detected experimentally (814 genes) the motif was demonstrated to be located at the expected distance (max. 60 nucleotides) from the TSS. As exemplified in the previously published primary regulon of SigX [19], we then defined a gene to be a member of the primary regulon of the *P*. *aeruginosa* sigma factors if it fulfilled at least two of the following three criteria: *i)* it exhibited sigma factor-dependent regulation of expression, *ii)* its promoter was enriched in ChIP-seq experiments and *iii)* its promoter contained a sigma factor binding motif. Detailed view on the intersections between the different approaches for the individual regulons is provided in S3 Fig., P values from the hypergeometric tests are listed in S7 Table. Based on these data, the primary *P*. *aeruginosa* sigma factor regulome is depicted in Fig. 3 (an interactive image is available at https://bactome.helmholtz-hzi.de/). S5 Table shows the promoter enrichment by means of ChIP-seq or motif search before applying our criteria for definition of primary regulons. S6 Table lists the discrete sets of genes belonging to the individual primary sigma factor regulons as defined above. The primary sigma factor regulome covers 2553 genes (43% of the genome) including 598 genes of unknown function. This number represents the most significant candidates which were obtained by stringent threshold settings. Due to the low conservation of the RpoD binding motif and no RpoD RNA-seq data, the RpoD regulon (encompassing 686 genes) is probably significantly underestimated.

**Fig 4:**
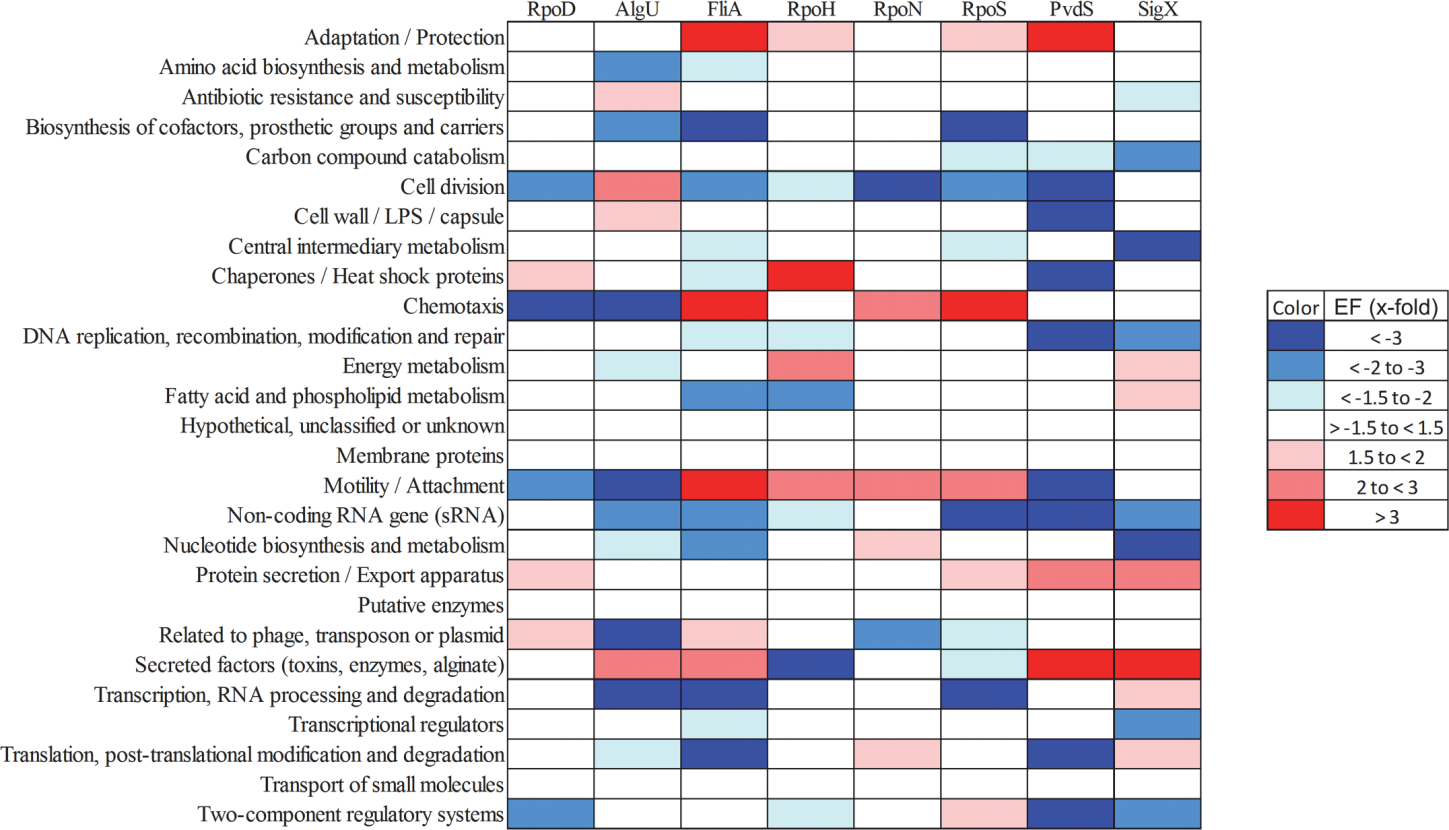
Functional characterization of primary sigma factor networks. The PseudoCAP annotation [55] was used to categorize the members of the sigma factor networks (Fig. 3, S6 Table). For each category the enrichment factor based on the prevalence of the specified class in the regulon compared to the whole genome was calculated. The P values of the enriched categories are provided in S8 Table.

Of note, meeting two out of the three of the criteria to define the sigma factor regulons decreased the regulon size of e.g. RpoH from 268 when meeting just one of the criteria (RNA–seq) (Table 1) to 96 (Table 2). However, on the other hand the regulon sizes of RpoN, AlgU and RpoS even increased, indicating that ChIP-seq in combination with a motif scan uncovered additional sigma factor binding sites. The validity of the selection criteria was further verified by functionally categorizing the members of each primary sigma factor regulon by the use of the PseudoCAP annotation [55]. The results are summarized in Fig. 4 (the enrichment values and their P values are listed in S8 Table). As expected, the AlgU regulon comprises genes of alginate biosynthesis and cellular homeostasis [59–63], the motility sigma factor FliA influences genes involved in chemotaxis and motility [64,65] and PvdS directs the pyoverdine biosynthesis genes [66] which are assigned to the category adaptation/protection. The heat-shock sigma factor RpoH governs the gene expression of chaperones and heat-shock proteins [67], while RpoN controls genes of nitrogen metabolism, chemotaxis, motility and attachment [68–70]. The stationary phase sigma factor RpoS regulates quorum sensing genes as well as genes involved in general adaptation processes [71,72]. A more detailed description of the individual regulons is provided in the supplementary material (S1 Text).

**Table 2:** Quantitative analysis of primary sigma factor regulons.

Regulon size^a^	867	680	347	341	316	272	228	84	28	26	18
Sigma factor	RpoD	RpoN	SigX	AlgU	FliA	RpoS	RpoH	PvdS	FecI2	FecI	FpvI
FpvI	1	0	2	0	2	0	0	1	0	0	**2**
FecI	8	1	1	0	0	0	0	0	0	**12**	0
FecI2	0	0	0	1	1	6	2	1	**15**	0	0
PvdS	7	2	6	1	4	5	2	**45**	1	0	1
RpoH	34	***26***	3	5	21	2	**96**	2	2	0	0
RpoS	28	***20***	9	12	15	**155**	2	5	6	0	0
FliA	9	***42***	18	20	**148**	15	21	4	1	0	2
AlgU	14	***51***	14	**202**	20	12	5	1	1	0	0
SigX	10	***39***	**215**	14	18	9	3	6	0	1	2
RpoN	52	**413**	39	51	42	20	26	2	0	1	0
RpoD	**686**	52	10	14	9	28	34	7	0	8	1

The total sizes of the primary regulons are listed at the top. Values in the colored cells show the numbers of genes present uniquely within one sigma factor regulon. The intersection values show how many genes were present in two regulons. The numbers in italic list the top 5 direct alternative sigma factor cross-talks.

^a^ The regulon size is the sum of all genes defined to belong to the primary regulon of the sigma factor using our criteria described in Materials and Methods.

### Crosstalk among sigma factor-associated networks

Beyond the assignment of genes to specific sigma factor regulons, our experimental design allowed us to define sets of genes that are affected by more than one sigma factor. We were able to assign as many as 1149 genes (61.6% of the primary alternative sigma factor regulome) to one distinct sigma factor regulon. Whereas those genes were exclusively affected by one sigma factor and did not participate in sigma factor crosstalk, 401 genes belonged to the primary regulon of more than one sigma factor (direct crosstalk) and 317 genes belonged to the primary regulon of one sigma factor, but were additionally affected on the transcriptional level by the activity of a second alternative sigma factor (indirect crosstalk) (Table 2). Both, the primary alternative sigma factor regulon and the RNA-seq data, revealed that all alternative sigma factors showed auto-regulation which is well-known for ECF sigma factors [16]. However, cross-talk among the sigma factors was very limited. We found only a direct impact of AlgU on *rpoH* expression, while indirect cross talk was identified between RpoH and *algU* as well between FpvI and *fecI2*. These results corroborate the finding of insulated sigma factor networks.

**Fig 5:**
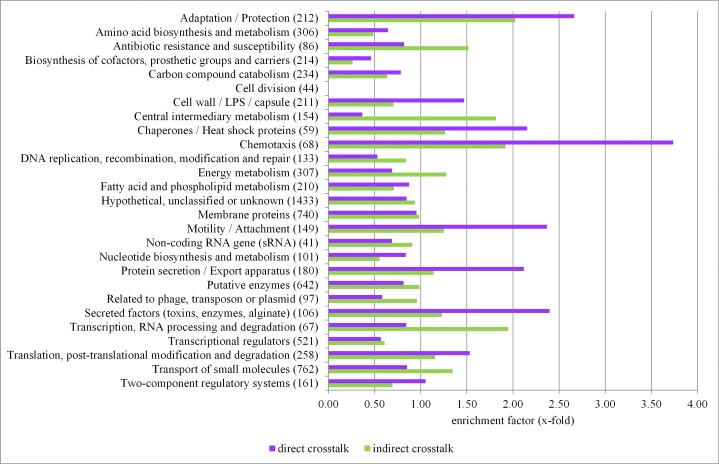
Functional profiling of the crosstalk among sigma factors in *P*. *aeruginosa*. The PseudoCAP annotation [55] was used to categorize the members of the direct (violet bars) and indirect (light green bars) sigma factor crosstalk. The enrichment of specific gene classes is displayed. The P values of the enriched categories are provided in S10 Table.

Direct crosstalk was mainly found to involve genes of the more complex functional categories adaptation/protection, chaperones/heat shock proteins, chemotaxis, motility/attachment, protein secretion and secreted factors (Fig. 5). There was also a preference of sigma factor combinations within the direct crosstalk. Direct crosstalk with RpoN clearly played the most dominant role (S2 Fig.). In total, 183 out of the 401 genes affected by direct crosstalk were found to be activated by RpoN in combination with either AlgU (51 genes), FliA (43 genes), SigX (40 genes), RpoH (26 genes) or RpoS (23 genes).

Functional profiling of the genes involved in the indirect crosstalk revealed that there was an enrichment of genes involved in central metabolic and cellular processes (Fig. 5 and S9 Table), indicating that cells are able to fine-tune expression levels of the most critical genes under various conditions via the activity of diverse sigma factors. The statistical significance of enrichment of individual PseudoCap classes has been addressed in S10 Table.

## Discussion

Gene expression is controlled by a complex regulatory system that makes it possible for cells to fine-tune their activity in response to changing environments. In bacteria, transcription initiation represents a major regulatory target which enables bacterial adaptation to challenging conditions and expression of virulence and pathogenicity. More recently the regulatory roles of sigma factors have gained increasing attention [19,25–27,29] as they provide promoter recognition specificity to the RNA polymerase core enzyme [12]. To date, up to 26 sigma factors have been described in *P*. *aeruginosa*, including 21 ECF sigma factors [14–16]. They play a crucial role in the transmission of extracellular signals to the cytoplasm and the initiation of a timely response to the specific extracellular conditions. The impact of individual alternative sigma factors on gene expression could be linked to bacterial virulence and pathogenicity [15,19,73–75].

The use of DNA microarrays and more recently RNA-seq approaches enabled the identification of transcriptional regulons on a genomic scale. Here, we describe the use of transcription profiling and ChIP-seq/chip to define the primary regulons of various alternative *P*. *aeruginosa* sigma factors and with this to set the stage for a very flexible experimental exploration of their functional states by transcriptional profiling under various physiological conditions.

Both transcriptional profiling as well as Chip-seq/chip were used in this study to determine the genome-wide targets of the various sigma factors. While transcriptional profiling determines the outcome of regulatory events for all genes within an operon, ChIP-seq identifies the protein-DNA interactions in the promoter region that determine these events. All changes in global RNA levels are recorded by transcriptional profiling regardless of whether those changes are directly due to the activity of the sigma factor or are a result of indirect effects. On the other hand binding of a transcription factor to its promoter target might not be associated with changes in RNA levels and some binding sites are located between divergently transcribed genes making it impossible to assign called peaks to respective promoter regions and thus to predict which gene might be regulated by sigma factor binding. The high gene density and the broad peaks of RNA-polymerase associated regulators like sigma factors lead to reduction of the strand-specificity. E. g. in the analysis of sigma factor networks in *E*. *coli* [76] the strand-specificity amounted to 69%. ChIP-seq is furthermore strongly dependent on an appropriate antibody. In this study, we provided the sigma factor genes fused to octahistidine-tag *in trans* in the PA14 wild-type strain and used a ChIP-grade antibody. We selected the his-tag because it generally does not impacts the structure of a protein [77] and it is less sensitive to formaldehyde-mediated crosslinking as compared to other tags which comprise lysine and arginine residues [78] [79].

In this study we complemented our RNA- and ChIP-seq approach with a global motif scan of *de novo* discovered binding motifs and applied very stringent threshold settings and rigorous statistical testing to define 2553 genes (43% of the genome) to belong to a sigma factor regulon. Those genes fulfilled at least 2 of the following 3 criteria: 1) they exhibited sigma factor-dependent regulation of expression; 2) their promoter was enriched in ChIP-seq experiments; 3) their promoter contained a sigma factor binding motif. Our results clearly demonstrate that especially when a combination of ChIP-seq and RNA-seq data are used to define primary regulons very robust information on transcriptional regulatory systems can be achieved. We found genome sequences of many previously described sigma factor-regulated genes to be enriched in each of the 10 alternative sigma factor regulons. They comprise a wide range of gene functions involved in sensing and responding to various conditions in the membrane, periplasm and extracellular environment, most of which have been implicated to play major roles in adaptation processes not only in *P*. *aeruginosa*, but also in other bacterial species. Furthermore, the validity of our selection criteria seems to be assured. Using a combinational approach we were able to identify a *de novo* consensus binding motif for every sigma factor and most of the promoter regions harbored only a unique sigma factor binding site.

In this study, we furthermore quantified the relative contribution of the 10 alternative sigma factors to the overall transcriptome plasticity of *P*. *aeruginosa* with the aim to uncover the architecture of the sigma factor regulons and to gain a more comprehensive understanding of the transcriptional network in this opportunistic pathogen. We found 67.8% of the genes of the PA14 genome to be affected by inactivation and/or *in trans* expression of the 10 alternative sigma factors. This is highly conform to a previously published impact of sigma factors on the transcriptome variance of *B*. *subtilis* (66%) as recorded under overall 104 different environmental conditions [80]. Furthermore, sigma factor regulatory network reconstructions in *B*. *subtilis* [80] revealed a highly modular structure of the various alternative sigma factor regulons as we observed here for *P*. *aeruginosa*. The interplay between four sigma factor regulatory networks was also analyzed in great detail in *G*. *sulfurreducens* [81] by the use of ChIP-chip/ChIP-seq approaches and transcriptional profiling of the wild-type under different growth conditions. Again, the operational state analysis showed a highly modular organization of the sigma factor networks.

This modular structure was not only reflected in the limited overlap of the primary alternative sigma factor regulons (direct crosstalk) but also become apparent when analyzing the sigma factor dependent transcriptional profiles. While the indirect crosstalk was preferentially assigned to central metabolic and cellular processes, the direct crosstalk was mainly found to involve genes of the functional categories adaptation/protection, chaperones/heat shock proteins, chemotaxis, motility/attachment, protein secretion and secreted factors. Obviously, complex processes such as chemotaxis and motility/attachment constitute higher-level functions which need the direct connection of diverse functional modules [82]. In line with this finding, a comprehensive analysis of the flagellar biosynthesis in *P*. *aeruginosa* revealed a four level hierarchy of transcriptional regulation involving RpoN and FliA as well as further transcriptional regulators [83]. In this study, the analysis of the most frequent sigma factor combinations uncovered RpoN as the central player within the sigma factor crosstalk, a role that can be attributed to numerous features. First, RpoN is widely distributed in the kingdom of bacteria in contrast to other alternative sigma factors [84]. Second, our results show that RpoN is the alternative sigma factor with the largest impact on global gene expression (680 genes) and is only outnumbered by the housekeeping sigma factor RpoD (867 genes). Third, *rpoN* is expressed constitutively and no anti-sigma factor for RpoN has been reported. This is of particular interest since even for the housekeeping sigma factor RpoD an anti-sigma factor has been identified [85]. Moreover, RpoN-dependent transcription is controlled by numerous co-activators allowing the modulation of RpoN activity [86]. Finally, RpoN has been shown to be involved in numerous functions from metabolism [68,87] to motility [69,70] to virulence [88,89].

In conclusion, the analysis of the architecture of the alternative sigma factor network in the opportunistic pathogen *P*. *aeruginosa* uncovered a highly modular structure with only limited crosstalk among alternative sigma factor regulons that are robustly activated in response to diverse forms of external stress. This is important since the survival of living systems critically relies on the robustness of essential modules and their insensitivity to many environmental and genetic perturbations. Our data support the view that widespread modularity exhibiting a self-contained activity guarantees robustness of biological networks in a noisy environment and thus provides bacteria with a framework to function adequately in their environment. At the same time we found connectivity of sigma factor modules to build up higher-level functions thus orchestrating complex cellular processes. Knowledge on the entire genomic suite of sigma factor binding sites throughout the *P*. *aeruginosa* genome will set the stage for a very flexible experimental exploration of their functional states by transcriptional profiling under various physiological conditions.

## Supporting Information

S1 Fig Sigma factor-dependent promoter activity studies in *P*. *aeruginosa*. The promoter activity of reporter strains based on selected sigma factor target promoter-*luxCDABE* fusions was determined by bioluminescence assays. The fold change of the reporter strain to the corresponding control strain is displayed including standard deviation. * P value <0.05, ** P value < 0.01 and *** P value < 0.001.
(TIF)

S2 Fig The TOP 10 sigma factor combinations within the direct sigma factor crosstalk in *P*. *aeruginosa*. The contribution of alternative sigma factors to the 10 most abundant sigma factor combinations within the direct crosstalk is illustrated. The five most dominant sigma factor combinations that cooperatively regulated the expression of genes were RpoN-AlgU, RpoN-FliA, RpoN-SigX, RpoN-RpoH and RpoN-RpoS which underlines the great significance of RpoN in sigma factor crosstalk.
(TIF)

S3 Fig Venn diagrams depicting the overlap between ChIP-seq, RNA-seq and motif search promoter hits for each sigma factor. The Venn diagrams show the intersections between the different approaches, the overlap significance was assessed by hypergeometric test. We included genes from the intersections in the primary regulons only if the P values of overlaps were maximally 0.05. Full details on the calculated P values are available in S7 Table.
(TIF)

S1 Text Characterization of primary sigma factor regulons. (DOCX)

S1 Table Primer, vectors and strains used in this study. (XLSX)

S2 Table Sigma factor-dependent gene expression according to mRNA profiling (OVR vs. WT + empty vector). (XLSX)

S3 Table Sigma factor-dependent gene expression according to mRNA profiling (KO vs. WT). (XLSX)

S4 Table Prediction of transcriptional units and updated PseudoCAP annotation. (XLSX)

S5 Table Sigma factor-dependent enrichment of promoter regions by ChIP-seq and motif search. (XLSX)

S6 Table Members and composition of the primary sigma factor regulons. (XLSX)

S7 Table Statistical analyses on the definition of primary sigma factor regulons. (XLSX)

S8 Table Pseudocap enrichment of functional classes in the sigma factor regulons. (XLSX)

S9 Table Members and composition of the sigma factor crosstalk. (XLSX)

S10 Table Pseudocap enrichment of functional classes in the direct and indirect crosstalk. (XLSX)
