# Supplementary figures and images for: Elucidation of Sigma Factor-Associated Networks in Pseudomonas aeruginosa Reveals a Modular Architecture with Limited and Function-Specific Crosstalk
Source: PLoS Pathog. 2015 Mar 17;11(3):e1004744. doi: 10.1371/journal.ppat.1004744 (PMC4362757; doi:10.1371/journal.ppat.1004744)

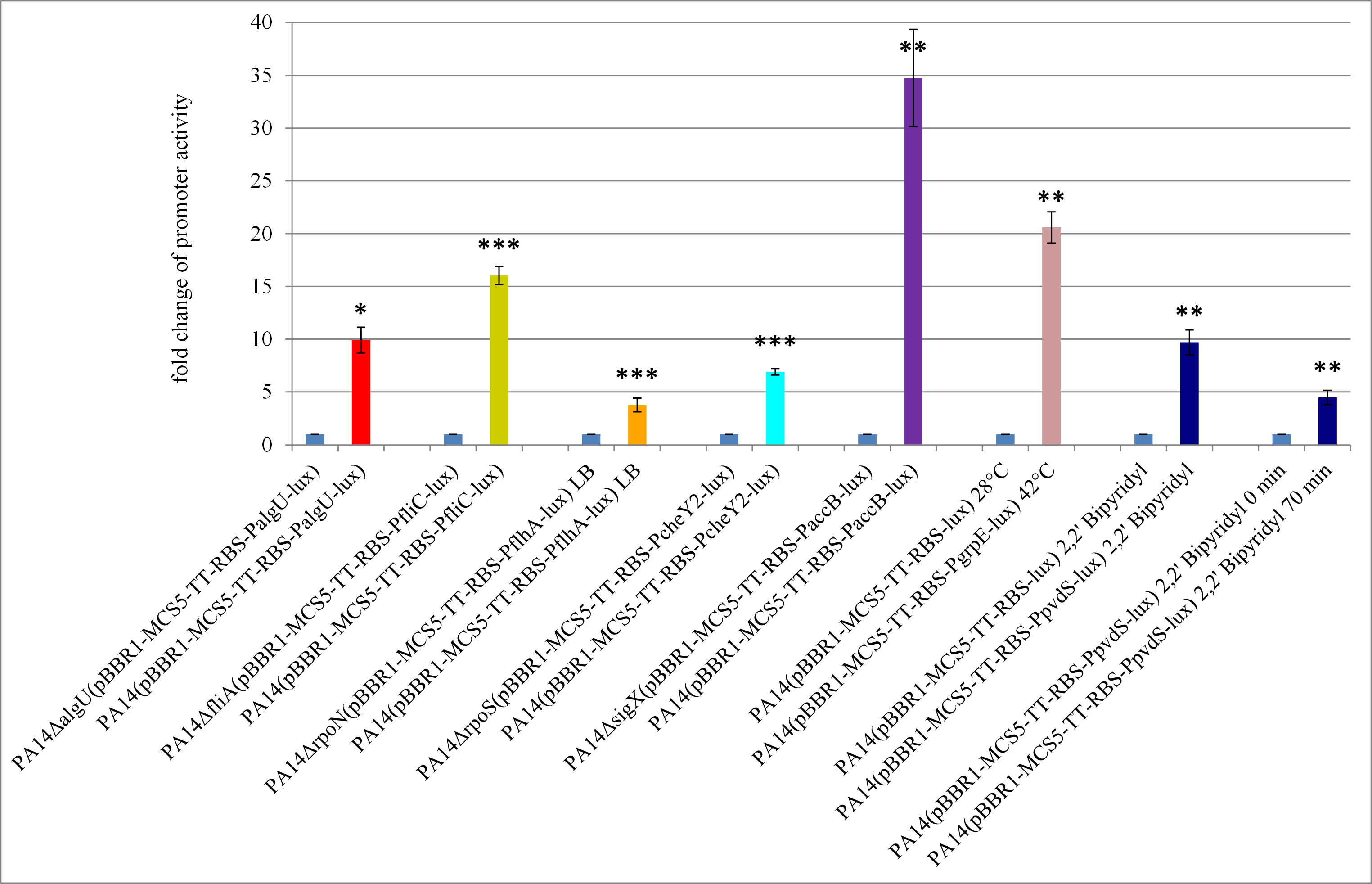

Supplement: S1 Fig — The promoter activity of reporter strains based on selected sigma factor target promoter-luxCDABE fusions was determined by bioluminescence assays. The fold change of the reporter strain to the corresponding control strain is displayed including standard deviation. * P value <0.05, ** P value < 0.01 and *** P value < 0.001. (TIF) [file ppat.1004744.s001.tif]

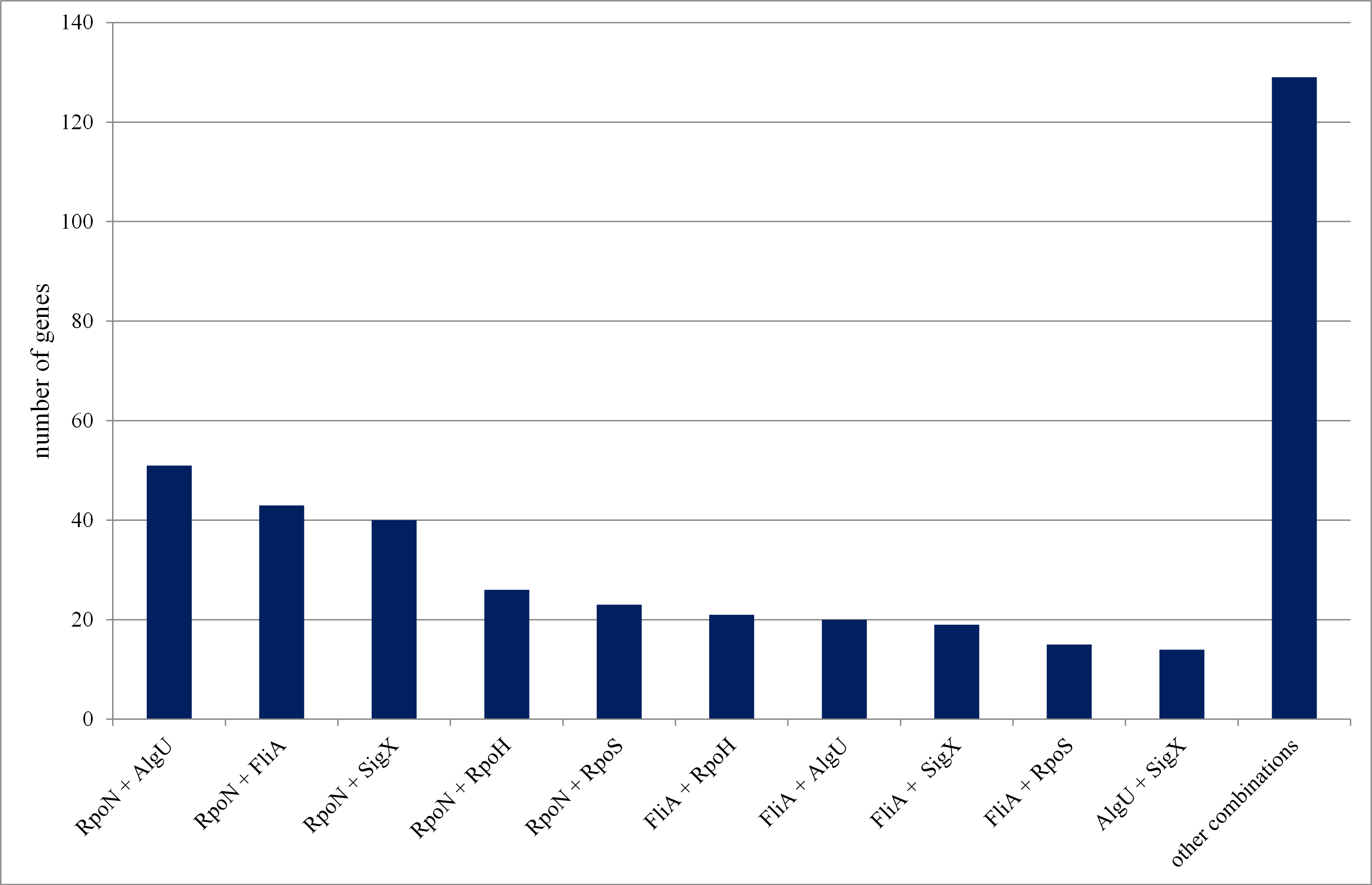

Supplement: S2 Fig — The contribution of alternative sigma factors to the 10 most abundant sigma factor combinations within the direct crosstalk is illustrated. The five most dominant sigma factor combinations that cooperatively regulated the expression of genes were RpoN-AlgU, RpoN-FliA, RpoN-SigX, RpoN-RpoH and RpoN-RpoS which underlines the great significance of RpoN in sigma factor crosstalk. (TIF) [file ppat.1004744.s002.tif]

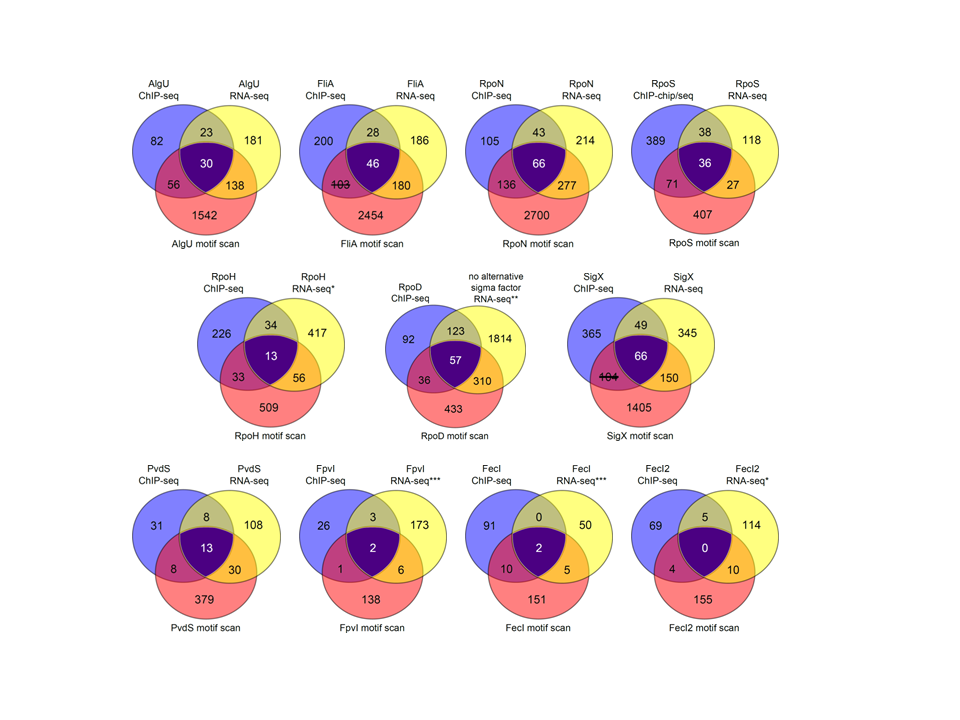

Supplement: S3 Fig — The Venn diagrams show the intersections between the different approaches, the overlap significance was assessed by hypergeometric test. We included genes from the intersections in the primary regulons only if the P values of overlaps were maximally 0.05. Full details on the calculated P values are available in S7 Table. (TIF) [file ppat.1004744.s003.tif]
