# Supplementary material for: Elucidation of Sigma Factor-Associated Networks in Pseudomonas aeruginosa Reveals a Modular Architecture with Limited and Function-Specific Crosstalk
Source: PLoS Pathog. 2015 Mar 17;11(3):e1004744. doi: 10.1371/journal.ppat.1004744 (PMC4362757; doi:10.1371/journal.ppat.1004744)
Supplement: S1 Text — (DOCX) [file ppat.1004744.s004.docx]

**Text S1. Characterization of primary sigma factor regulons**

**The primary AlgU regulon**

The ECF sigma factor AlgU is the key regulator of alginate biosynthesis [[1](#_ENREF_1),[2](#_ENREF_2)]. AlgU governs the general cell envelope stress response to various environmental challenges such as extreme heat-shock [[3](#_ENREF_3)], oxidative stress [[4](#_ENREF_4)], cell wall-inhibitory antibiotics [[5](#_ENREF_5)] and spaceflight-analogue conditions [[6](#_ENREF_6)] and is also involved in biofilm formation [[7](#_ENREF_7)]. The broad impact of AlgU on global gene expression is reflected by its primary regulon size of 341 genes organized in 247 TUs (Table 2). The AlgU binding motif was deduced from 49 promoter regions (Figure 2) and is similar to the previously proposed AlgU consensus sequence GAACTT-N_16-17_-TCtgA [[8](#_ENREF_8)]. In accordance with the known functions of AlgU, there is a strong to moderate gene enrichment in PseudoCAP categories which are associated with cell envelope integrity such as adaptation/protection, cell wall/LPS/capsule, antibiotic resistance and susceptibility and fatty acid and phospholipid metabolism (Figure 4). Prominent members of the primary AlgU regulon (Table S5) are the alginate genes *alg8-alg44-algKEGXL* and *algC* as well as the operon *algU*-*mucABCD*. Importantly, our regulon analysis shows both reported auto-regulation of *algU* [[2](#_ENREF_2)] and the regulation of the cytoplasmatic sigma factor *rpoH* [[9](#_ENREF_9)].

**The primary FliA regulon**

FliA is the alternative sigma factor which is linked to motility and thus orchestrates the expression of flagellar and chemotaxis genes in many bacterial species like *P. aeruginosa* [[10](#_ENREF_10)], *E. coli* [[11](#_ENREF_11)], *S. typhimurium* [[12](#_ENREF_12)] and *B. subtilis* [[13](#_ENREF_13)]. Accordingly, the functional profiling of the primary FliA regulon displays the strongly over-represented categories chemotaxis, motility/attachment and adaptation/protection (Figure 4). 31 out of 68 genes associated with chemotaxis were identified. More specifically, key chemotaxis genes that are targeted by FliA are the operon *cheY**-*cheAW*-PA14_02220- *cheR**-*cheD**-*cheB*, *ctpH**, *aer* as wells as *pctA*, *pctB*, *pctC*, *motAB*, *motCD*, while prominent detected motility and attachment genes are *fliEFGHIJ*, *fliC, fliD* and *flgL* as wells *pilMNOPQ* and *morA* (Table S5). In addition, our primary FliA regulon also includes *fliA* itself indicating auto-regulation of this non-ECF sigma factor as well as *flgM* which encodes the anti-sigma factor of FliA [[14](#_ENREF_14)]. The primary FliA regulon encompasses 316 genes (254 TUs) and its size is similar to the size of the alternative sigma factors AlgU and SigX (Table 2). The identified FliA motif (Figure 4) which relies on 35 promoter regions shows high similarity with the proposed FliA consensus sequence TCAAG-t-N_12-13_-GCCGATA in *P. putida* [[15](#_ENREF_15)].

**The primary PvdS regulon**

PvdS is the major ECF sigma factor for iron-acquisition under iron-starvation conditions by orchestrating the production of the siderophore pyoverdine [[16](#_ENREF_16)]. Accordingly, key genes like pvdA, PA14_33610-pvdJD, pvdE, pvdF, PA14_33730-pvdNO, pvdGL and pvdP as well as the stringent starvation genes sspAB were strongly enriched within the PseudoCAP class adaptation/protection (Figure 4). PvdS has also been reported to be involved in regulation of virulence-associated genes [[17](#_ENREF_17),[18](#_ENREF_18)] and our primary PvdS regulon includes the endoprotease PrpL [[19](#_ENREF_19)], the exoenzyme ExoT [[20](#_ENREF_20)] and the pyocin killing protein PyoS3A (Table S5). Further prominent regulon members are *pvdS* itself and the transcriptional regulator *toxR*. Strikingly, the overall enrichment profile of the primary PvdS regulon is characterized by the entire absence of seven functional classes. The high specificity is further underlined by the regulon size of only 84 genes corresponding to 59 TUs (Table 2). The uncovered bipartite PvdS binding motif (Figure 2) is in good agreement with the previously published consensus sequence TAAAT(A/T)-N_15_-CGTT(C/T)(T/A) in *P. syringae* [[21](#_ENREF_21)].

**The primary RpoH regulon**

The alternative sigma factor RpoH was discovered in the context of the heat-shock response in *E. coli* [[22](#_ENREF_22),[23](#_ENREF_23)] and subsequently identified in *P. aeruginosa* [[24](#_ENREF_24),[25](#_ENREF_25)] and many other Gram-negative bacteria [[26](#_ENREF_26),[27](#_ENREF_27)]. The RpoH-dependent response to elevated temperatures is mediated by heat-shock proteins, chaperons and specific proteases. These important effectors are also required for an adequate response to other stress conditions suggesting that RpoH plays a central role in the direction of maintaining cytoplasmatic homoestasis [[23](#_ENREF_23),[28](#_ENREF_28)]. Accordingly, the primary RpoH regulon comprises the key genes *groES*-*groEL*, *grpE*-*dnaK*, *dnaJ-dapB*, *ibpA*, *lon*, *clpB*, *clpX*, *htpG*, and *hslVU* which contribute to a strong gene enrichment of the class chaperones/heat shock proteins (Figure 4, Table S5). Furthermore, we could demonstrate auto-regulation of *rpoH*. Further prominent regulon members are the post-transcriptional regulator *hfq* and the cell division protein *ftsH*. The primary RpoH regulon comprises only 228 genes (136 TUs) and thus is only ranked seventh in size (Table 2). The reported RpoH consensus sequence cTTGAA-N_13-16_-(a/c)CCATat(a/t) in *V. cholera* [[29](#_ENREF_29)] is in agreement with our elucidated motif which is based on a selection of eleven promoter regions (Figure 2).

**The primary RpoN regulon**

RpoN was shown to impact on the transcriptional control of nitrogen-regulated promoter [[30](#_ENREF_30),[31](#_ENREF_31)]. Accordingly, key genes like *rpoN* itself, *ntrBC*, *glnA*, *glnK*-*amtB* as well as *nirBD*, *nasA*, *nasST* and the operon *nosRZDFYL* are prominent members of this primary regulon. RpoN also controls the regulation of genes assigned to nucleotide biosynthesis and metabolism (Figure 4). Moreover, RpoN is required for the expression of flagellin and pilin genes [[32](#_ENREF_32),[33](#_ENREF_33)]. Consistently, our primary regulon includes genes of the *fli* and *flg* cluster namely *fliD*, *fliEFGHIJ*, *fliL*MNOPQR* and *fliS* as well as *flgA*, *flgBCDE* and *flgFGHIJKL* (Table S5). RpoN also controls 53 genes which are assigned to translation, post-translational modification and degradation like the cluster *rpl*, *rps* and *rpm*. Strikingly, RpoN shows an enrichment of non-coding RNA genes. Further RpoN targets are the virulence factor regulator gene *vfr*, the anaerobically-induced genes *anr* and *oprE* [[34](#_ENREF_34)] as well as quorum sensing related genes *rhlAB* and *rhlR*. With 680 genes (522 TUs) the primary RpoN regulon has the largest size among all alternative sigma factors (Table 2). Our identified RpoN motif (Figure 2) confirms the proposed consensus sequence TGGca-N_4-5_-ttGCaa which was deduced from ChIP-chip experiments in *E. coli* [[35](#_ENREF_35)].

**The primary RpoS regulon**

RpoS was firstly identified in *E. coli* during carbon-starvation-induced entry into the stationary growth phase and is required for an adequate response to several stressors like acidicity, H_2_O_2_ and heat shock [[36](#_ENREF_36)]. Furthermore, RpoS plays an important role within the quorum sensing circuit [[37](#_ENREF_37),[38](#_ENREF_38)]. In line with a previous study by Schuster and colleagues [[39](#_ENREF_39)], our integrative approach (Figure 4, Table S5) identified strong gene enrichments within the functional classes chemotaxis (10 genes) and two-component regulatory systems (15 genes). In addition and according to the general role of RpoS, the category adaptation/protection shows significant gene enrichment. Further interesting regulon members are the origin of replication operon *dnaAN*-*recF*-*gyrB,* the cytochrome-c related operons *coxBA*G*-*coIII* and *napEFDABC* as well as genes of the succinate dehydrogenase subunits *sdhCDAB*, the DNA repair proteins *recO* and *radC*. This highlights the contribution of RpoS to switching energy metabolism and supporting DNA replication, recombination, modification and repair. RpoS also targets itself as well as the transcriptional regulators *rhlR* and *rsmA*. The primary RpoS regulon size of 272 genes (172 TUs) is surprisingly small (Table 2). The RpoS motif comprises a -10 element only and our elucidated motif (Figure 2) confirms the proposed consensus sequence CTATACT [[39](#_ENREF_39)].

**The primary SigX regulon**

The ECF sigma factor SigX was discovered in the context of its regulatory effect on the major outer membrane protein OprF and its role in osmolarity [[40](#_ENREF_40)]. With 347 genes (265 TUs) the primary SigX regulon size is ranked third behind RpoD and RpoN (Table 2). Despite our integrative approach, our SigX motif shows only low sequence conservation (Figure 2). Interestingly, there is a large overlap of over-represented PseudoCAP categories with the SigX regulon study by Gicquel and colleagues [[41](#_ENREF_41)] (Figure 4, Table S5). In detail, there is a strong enrichment for genes involved in secretion pathways like members of the type III secretion system *pscBCDEFGHIJKL*, *exsA*, *exsC*-PA14_42410-*exsB* and the two phenazine cluster *phzB1C1D1E1F1G1* and *phzB2E2F2G2* as well as genes linked to energy metabolism like the cytochrome-c oxidase subunits *coxBA*G*-*coIII* and the ATP synthase chains *atpEFHAGDC*. Importantly, genes associated with fatty acid and phospholipid metabolism like *accA*, *accBC*, *fabAB*, *fabD*, *fabH2*, *fabH-2**, *fabZ* and the methyltransferase gene *pmtA* were preferentially targeted by SigX. SigX also controls 30 genes linked to translation, post-translational modification and degradation.

**The primary RpoD regulon**

In 1969, RpoD was identified as a sigma factor which is mandatory to stimulate transcription by the RNA polymerase in *E. coli* [[42](#_ENREF_42)]. As the principal sigma factor, RpoD is involved in the expression of genes associated with housekeeping processes [[43](#_ENREF_43)]. Accordingly, prominent gene members of the identified primary regulon are the chromosomal replication initiator protein *dnaA*, DNA polymerase III *dnaN*, the DNA replication and repair protein *recF* the DNA gyrase subunits, *gyrB* and *gyrA*, the transcriptional regulator *dnr*, the regulator of secondary metabolites *rsmA*, the signal recognition particle protein *ffh*, the ribosome modulation factor *rmf*, the transcription-repair coupling factor *mfd*, the ribonuclease E *rne*, the electron transfer flavoproteins *etfA* and *etfB*, the fatty acid oxidation complex *fadA* and *fadB*, the translation initiation factor IF-1 *infA*, the peptide chain release factor 1 *prfA*, the putative host factor-I protein *hfq*, the ferric uptake regulation protein *fur*, the redoxin proteins *fdxA*, *grx* and *trxA* and the small regulatory RNAs *rsmY*, *rsmZ*, *rgsA*, *phrS*, *phrY*, P32 and *ssrS* as well as *rpoD* itself. The significance of RpoD is further underlined by its large impact on global regulation of gene expression in respect to quantity amounting to 867 genes corresponding to 526 TUs (Table 2 and S5) as well as in respect to quality reflected by its broad functional profile (Figure 4). Transcription of *rpoD* is most dominant during the exponential growth phase and driven from two promoters, one constitutive active and one transiently induced upon heat shock [[44](#_ENREF_44),[45](#_ENREF_45)]. In contrast to RpoH, the RpoD-dependent over-representation of the category chaperones/heat shock proteins is mainly related to the high amount of chaperones like the isomerases SurA, PpiD, Fkl*, FkbP-1*, PpiC1, DsbB, DipZ2*, DsbG and PA14_32600. This study reveals that RpoD-dependent gene regulation provides the basal import/export infrastructure by controlling the general secretion pathway operons *xcpPQ* and *xcpRSTUVWXYZ* as well as *secG*, *secB*, *secDF*, *tatBC* and the cluster of the type II and III secretion system *hplRSTUVW** and *popN*-*pcr1234**-*pcrDRGVH*-*popBD* respectively. Interestingly, genes which are related to phage, transposon or plasmid show strong preference for RpoD indicating phage adaptation to the host. Consistent with its housekeeping function, RpoD preferentially targets genes linked to amino acid biosynthesis and metabolism, cell wall components, secreted factors and translation, post-translational modification and degradation. Surprisingly, our RpoD regulon analysis features less genes associated with the cell cycle than anticipated. Finally, the highly specialized functional classes chemotaxis and motility/attachment governed by the sigma factors FliA, RpoN and RpoS as well as two-component regulatory systems mainly directed by RpoS are found to be strongly under-represented within the primary RpoD regulon.

**The primary FpvI, FecI and FecI2 regulon**

The primary regulon sizes of FpvI (12 genes), FecI (17 genes) and FecI2 (19 genes) are the smallest among all investigated sigma factors (Table 2 and S5). FpvI plays an important role in pyoverdine-mediated iron-acquisition by regulating the gene expression of the ferric pyoverdine receptor *fpvA* [[46](#_ENREF_46)]. Accordingly, key members of the primary FpvI regulon are *fpvI* itself, its corresponding receptor *fpvA*, the L-ornithine N5-oxygenase *pvdA*, the haem uptake outer membrane receptor *hasR* and the small non-coding RNA *prrF1*. FecI (PA14_13460) is the only iron-starvation sigma factor known in *E. coli* and has been assigned to the uptake of ferric citrate [[47](#_ENREF_47)]. Further prominent players of the primary FecI regulon are *hasR*, *pvdA*, *cirA**, *oprG*, *potD* and *ybeJ*.* The sigma factor FecI2 (PA14_27690) is commonly present in *P. aeruginosa*, however not in all strains e.g. it is missing in the PAO1 type strain [[48](#_ENREF_48)]. The FecI2 regulon encompasses iron-starvation and virulence-related genes such as *fecI2* itself, *pchR*, *hasR*, *hasAp*, *phuR*, *hxuC**, *fecA** and *pvdS*. The identified motifs of all three sigma factors are AT-rich (Figure 2). This result is particularly characteristic for iron-starvation target promoters which harbor the so-called ‘iron box’ [[49](#_ENREF_49)].

**References**

1. Schurr MJ, Martin DW, Mudd MH, Hibler NS, Boucher JC, et al. (1993) The algD promoter: regulation of alginate production by Pseudomonas aeruginosa in cystic fibrosis. Cell Mol Biol Res 39: 371-376.

2. Hershberger CD, Ye RW, Parsek MR, Xie ZD, Chakrabarty AM (1995) The algT (algU) gene of Pseudomonas aeruginosa, a key regulator involved in alginate biosynthesis, encodes an alternative sigma factor (sigma E). Proc Natl Acad Sci U S A 92: 7941-7945.

3. Schurr MJ, Yu H, Boucher JC, Hibler NS, Deretic V (1995) Multiple promoters and induction by heat shock of the gene encoding the alternative sigma factor AlgU (sigma E) which controls mucoidy in cystic fibrosis isolates of Pseudomonas aeruginosa. J Bacteriol 177: 5670-5679.

4. Yu H, Schurr MJ, Deretic V (1995) Functional equivalence of Escherichia coli sigma E and Pseudomonas aeruginosa AlgU: E. coli rpoE restores mucoidy and reduces sensitivity to reactive oxygen intermediates in algU mutants of P. aeruginosa. J Bacteriol 177: 3259-3268.

5. Wood LF, Leech AJ, Ohman DE (2006) Cell wall-inhibitory antibiotics activate the alginate biosynthesis operon in Pseudomonas aeruginosa: Roles of sigma (AlgT) and the AlgW and Prc proteases. Mol Microbiol 62: 412-426.

6. Crabbe A, Pycke B, Van Houdt R, Monsieurs P, Nickerson C, et al. (2010) Response of Pseudomonas aeruginosa PAO1 to low shear modelled microgravity involves AlgU regulation. Environ Microbiol 12: 1545-1564.

7. Bazire A, Shioya K, Soum-Soutera E, Bouffartigues E, Ryder C, et al. (2010) The sigma factor AlgU plays a key role in formation of robust biofilms by nonmucoid Pseudomonas aeruginosa. J Bacteriol 192: 3001-3010.

8. Firoved AM, Boucher JC, Deretic V (2002) Global genomic analysis of AlgU (sigmaE)-dependent promoters (sigmulon) in Pseudomonas aeruginosa and implications for inflammatory processes in cystic fibrosis. J Bacteriol 184: 1057-1064.

9. Schurr MJ, Deretic V (1997) Microbial pathogenesis in cystic fibrosis: co-ordinate regulation of heat-shock response and conversion to mucoidy in Pseudomonas aeruginosa. Mol Microbiol 24: 411-420.

10. Starnbach MN, Lory S (1992) The fliA (rpoF) gene of Pseudomonas aeruginosa encodes an alternative sigma factor required for flagellin synthesis. Mol Microbiol 6: 459-469.

11. Arnosti DN, Chamberlin MJ (1989) Secondary sigma factor controls transcription of flagellar and chemotaxis genes in Escherichia coli. Proc Natl Acad Sci U S A 86: 830-834.

12. Ohnishi K, Kutsukake K, Suzuki H, Iino T (1990) Gene fliA encodes an alternative sigma factor specific for flagellar operons in Salmonella typhimurium. Mol Gen Genet 221: 139-147.

13. Mirel DB, Chamberlin MJ (1989) The Bacillus subtilis flagellin gene (hag) is transcribed by the sigma 28 form of RNA polymerase. J Bacteriol 171: 3095-3101.

14. Frisk A, Jyot J, Arora SK, Ramphal R (2002) Identification and functional characterization of flgM, a gene encoding the anti-sigma 28 factor in Pseudomonas aeruginosa. J Bacteriol 184: 1514-1521.

15. Rodriguez-Herva JJ, Duque E, Molina-Henares MA, Navarro-Aviles G, van Dillewijn P, et al. (2010) Physiological and transcriptomic characterization of a fliA mutant of Pseudomonas putida KT2440. Environmental Microbiology Reports 2: 373-380.

16. Cunliffe HE, Merriman TR, Lamont IL (1995) Cloning and characterization of pvdS, a gene required for pyoverdine synthesis in Pseudomonas aeruginosa: PvdS is probably an alternative sigma factor. J Bacteriol 177: 2744-2750.

17. Lamont IL, Beare PA, Ochsner U, Vasil AI, Vasil ML (2002) Siderophore-mediated signaling regulates virulence factor production in Pseudomonas aeruginosa. Proc Natl Acad Sci U S A 99: 7072-7077.

18. Beare PA, For RJ, Martin LW, Lamont IL (2003) Siderophore-mediated cell signalling in Pseudomonas aeruginosa: divergent pathways regulate virulence factor production and siderophore receptor synthesis. Mol Microbiol 47: 195-207.

19. Wilderman PJ, Vasil AI, Johnson Z, Wilson MJ, Cunliffe HE, et al. (2001) Characterization of an endoprotease (PrpL) encoded by a PvdS-regulated gene in Pseudomonas aeruginosa. Infect Immun 69: 5385-5394.

20. Ochsner UA, Johnson Z, Lamont IL, Cunliffe HE, Vasil ML (1996) Exotoxin A production in Pseudomonas aeruginosa requires the iron-regulated pvdS gene encoding an alternative sigma factor. Mol Microbiol 21: 1019-1028.

21. Swingle B, Thete D, Moll M, Myers CR, Schneider DJ, et al. (2008) Characterization of the PvdS-regulated promoter motif in Pseudomonas syringae pv. tomato DC3000 reveals regulon members and insights regarding PvdS function in other pseudomonads. Mol Microbiol 68: 871-889.

22. Grossman AD, Erickson JW, Gross CA (1984) The htpR gene product of E. coli is a sigma factor for heat-shock promoters. Cell 38: 383-390.

23. Erickson JW, Vaughn V, Walter WA, Neidhardt FC, Gross CA (1987) Regulation of the promoters and transcripts of rpoH, the Escherichia coli heat shock regulatory gene. Genes Dev 1: 419-432.

24. Allan B, Linseman M, MacDonald LA, Lam JS, Kropinski AM (1988) Heat shock response of Pseudomonas aeruginosa. J Bacteriol 170: 3668-3674.

25. Benvenisti L, Koby S, Rutman A, Giladi H, Yura T, et al. (1995) Cloning and primary sequence of the rpoH gene from Pseudomonas aeruginosa. Gene 155: 73-76.

26. Nakahigashi K, Yanagi H, Yura T (1995) Isolation and sequence analysis of rpoH genes encoding sigma 32 homologs from gram negative bacteria: conserved mRNA and protein segments for heat shock regulation. Nucleic Acids Res 23: 4383-4390.

27. Nakahigashi K, Yanagi H, Yura T (1998) Regulatory conservation and divergence of sigma32 homologs from gram-negative bacteria: Serratia marcescens, Proteus mirabilis, Pseudomonas aeruginosa, and Agrobacterium tumefaciens. J Bacteriol 180: 2402-2408.

28. Manzanera M, Aranda-Olmedo I, Ramos JL, Marques S (2001) Molecular characterization of Pseudomonas putida KT2440 rpoH gene regulation. Microbiology 147: 1323-1330.

29. Slamti L, Livny J, Waldor MK (2007) Global gene expression and phenotypic analysis of a Vibrio cholerae rpoH deletion mutant. J Bacteriol 189: 351-362.

30. Hunt TP, Magasanik B (1985) Transcription of glnA by purified Escherichia coli components: core RNA polymerase and the products of glnF, glnG, and glnL. Proc Natl Acad Sci U S A 82: 8453-8457.

31. Gussin GN, Ronson CW, Ausubel FM (1986) Regulation of nitrogen fixation genes. Annu Rev Genet 20: 567-591.

32. Ishimoto KS, Lory S (1989) Formation of Pilin in Pseudomonas-Aeruginosa Requires the Alternative Sigma-Factor (RpoN) of RNA-Polymerase. Proceedings of the National Academy of Sciences of the United States of America 86: 1954-1957.

33. Totten PA, Lara JC, Lory S (1990) The RpoN Gene-Product of Pseudomonas Aeruginosa Is Required for Expression of Diverse Genes, Including the Flagellin Gene. Journal of Bacteriology 172: 389-396.

34. Yamano Y, Nishikawa T, Komatsu Y (1998) Involvement of the RpoN protein in the transcription of the oprE gene in Pseudomonas aeruginosa. Fems Microbiology Letters 162: 31-37.

35. Zhao K, Liu M, Burgess RR (2010) Promoter and regulon analysis of nitrogen assimilation factor, sigma54, reveal alternative strategy for E. coli MG1655 flagellar biosynthesis. Nucleic Acids Res 38: 1273-1283.

36. Lange R, Hengge-Aronis R (1991) Identification of a central regulator of stationary-phase gene expression in Escherichia coli. Mol Microbiol 5: 49-59.

37. Latifi A, Foglino M, Tanaka K, Williams P, Lazdunski A (1996) A hierarchical quorum-sensing cascade in Pseudomonas aeruginosa links the transcriptional activators LasR and RhIR (VsmR) to expression of the stationary-phase sigma factor RpoS. Molecular Microbiology 21: 1137-1146.

38. Whiteley M, Parsek MR, Greenberg EP (2000) Regulation of quorum sensing by RpoS in Pseudomonas aeruginosa. J Bacteriol 182: 4356-4360.

39. Schuster M, Hawkins AC, Harwood CS, Greenberg EP (2004) The Pseudomonas aeruginosa RpoS regulon and its relationship to quorum sensing. Mol Microbiol 51: 973-985.

40. Brinkman FS, Schoofs G, Hancock RE, De Mot R (1999) Influence of a putative ECF sigma factor on expression of the major outer membrane protein, OprF, in Pseudomonas aeruginosa and Pseudomonas fluorescens. J Bacteriol 181: 4746-4754.

41. Gicquel G, Bouffartigues E, Bains M, Oxaran V, Rosay T, et al. (2013) The Extra-Cytoplasmic Function Sigma Factor SigX Modulates Biofilm and Virulence-Related Properties in. PLoS One 8: e80407.

42. Burgess RR, Travers AA, Dunn JJ, Bautz EK (1969) Factor stimulating transcription by RNA polymerase. Nature 221: 43-46.

43. Helmann JD, Chamberlin MJ (1988) Structure and function of bacterial sigma factors. Annu Rev Biochem 57: 839-872.

44. Fujita M, Tanaka K, Takahashi H, Amemura A (1994) Transcription of the principal sigma-factor genes, rpoD and rpoS, in Pseudomonas aeruginosa is controlled according to the growth phase. Mol Microbiol 13: 1071-1077.

45. Aramaki H, Fujita M (1999) In vitro transcription analysis of rpoD in Pseudomonas aeruginosa PAO1. FEMS Microbiol Lett 180: 311-316.

46. Redly GA, Poole K (2003) Pyoverdine-mediated regulation of FpvA synthesis in Pseudomonas aeruginosa: involvement of a probable extracytoplasmic-function sigma factor, FpvI. J Bacteriol 185: 1261-1265.

47. Ochs M, Angerer A, Enz S, Braun V (1996) Surface signaling in transcriptional regulation of the ferric citrate transport system of Escherichia coli: mutational analysis of the alternative sigma factor FecI supports its essential role in fec transport gene transcription. Mol Gen Genet 250: 455-465.

48. Potvin E, Sanschagrin F, Levesque RC (2008) Sigma factors in Pseudomonas aeruginosa. FEMS Microbiol Rev 32: 38-55.

49. de Lorenzo V, Wee S, Herrero M, Neilands JB (1987) Operator sequences of the aerobactin operon of plasmid ColV-K30 binding the ferric uptake regulation (fur) repressor. J Bacteriol 169: 2624-2630.
